# Susceptibility of *Anopheles gambiae* from Côte d’Ivoire to insecticides used on insecticide-treated nets: evaluating the additional entomological impact of piperonyl butoxide and chlorfenapyr

**DOI:** 10.1186/s12936-020-03523-y

**Published:** 2020-12-09

**Authors:** Bernard L. Kouassi, Constant Edi, Emmanuel Tia, Lucien Y. Konan, Maurice A. Akré, Alphonsine A. Koffi, Allassane F. Ouattara, Antoine Mea Tanoh, Pascal Zinzindohoue, Blaise Kouadio, McKenzie Andre, Seth R. Irish, Jennifer Armistead, Dereje Dengela, Ndombour G. Cissé, Cecilia Flatley, Joseph Chabi

**Affiliations:** 1 PMI VectorLink project, Abidjan, Côte d’Ivoire; 2 Swiss Center of Scientific Research in Côte d’Ivoire, Abidjan, Côte d’Ivoire; 3 Centre of Veterinary and Medical Entomology, Abidjan, Côte d’Ivoire; 4 National Institute of Public Hygiene, Abidjan, Côte d’Ivoire; 5 National Institute of Public Health/Pierre Richet Institute, Bouake, Côte d’Ivoire; 6 National Malaria Control Programme, Abidjan, Côte d’Ivoire; 7 U.S. President’s Malaria Initiative, USAID, Abidjan, Côte d’Ivoire; 8 U.S. President’s Malaria Initiative, Entomology Branch, U.S. Centers for Disease Control and Prevention, Atlanta, GA USA; 9 U.S. President’s Malaria Initiative, USAID, Washington, DC USA; 10 PMI VectorLink Project, Washington, DC USA

**Keywords:** Insecticide resistance, Pyrethroids, Piperonyl butoxide, Chlorfenapyr, *Anopheles gambiae*, Vector control, ITNs, Côte d’Ivoire

## Abstract

**Background:**

Pyrethroid-treated mosquito nets are currently the mainstay of vector control in Côte d’Ivoire. However, resistance to pyrethroids has been reported across the country, limiting options for insecticide resistance management due to the paucity of alternative insecticides. Two types of insecticide-treated nets (ITNs), ITNs with pyrethroids and the synergist piperonyl butoxide (PBO), and Interceptor®G2 nets, a net treated with a combination of chlorfenapyr and alpha-cypermethrin, are believed to help in the control of pyrethroid-resistant mosquitoes.

**Methods:**

The susceptibility of *Anopheles gambiae *sensu lato (s.l.) to pyrethroid insecticides with and without pre-exposure to PBO as well as to chlorfenapyr was investigated in fifteen sites across the country. Susceptibility tests were conducted on 2- to 4-day old adult female *An. gambiae* s.l. reared from larval collections. The resistance status, intensity, and effects of PBO on mortality after exposure to different concentrations of deltamethrin, permethrin and alpha-cypermethrin were determined using WHO susceptibility test kits. In the absence of a WHO-recommended standard protocol for chlorfenapyr, two interim doses (100 and 200 µg/bottle) were used to test the susceptibility of mosquitoes using the CDC bottle assay method.

**Results:**

Pre-exposure to PBO did not result in full restoration of susceptibility to any of the three pyrethroids for the *An. gambiae* s.l. populations from any of the sites surveyed. However, PBO pre-exposure did increase mortality for all three pyrethroids, particularly deltamethrin (from 4.4 to 48.9%). *Anopheles gambiae* s.l. from only one site (Bettie) were susceptible to chlorfenapyr at the dose of 100 µg active ingredient (a.i.)/bottle. At the dose of 200 µg (a.i.)/bottle, susceptibility was only recorded in 10 of the 15 sites.

**Conclusion:**

Low mosquito mortality was found for pyrethroids alone, and while PBO increased mortality, it did not restore full susceptibility. The vector was not fully susceptible to chlorfenapyr in one third of the sites tested. However, vector susceptibility to chlorfenapyr seems to be considerably higher than for pyrethroids alone or with PBO. These data should be used cautiously when making ITN procurement decisions, noting that bioassays are conducted in controlled conditions and may not fully represent field efficacy where the host-seeking behaviours, which include free-flying activity are known to enhance pro-insecticide chlorfenapyr intoxication to mosquitoes.

## Background

The recent malaria control success recorded in several malaria endemic countries is largely attributed to the scale up of vector control tools, particularly the implementation and high coverage of insecticide-treated nets (ITNs) and indoor residual spraying (IRS) [1]. Pyrethroid insecticides have been used for several years both for ITNs and IRS because of their effectiveness in killing mosquitoes and also for their repellent properties, providing users additional personal protection and safety during sleeping hours [2, 3]. Due to their low cost, long residual activity and safety, pyrethroids remain the recommended insecticides for treating ITNs [4]. Unfortunately, the spread of target-site insensitivity and metabolic resistance mechanisms against pyrethroid insecticides occurring in malaria vectors are a threat to vector control and could lead to resurgence of malaria in endemic countries [5–7].

Resistance to all the insecticides previously used in public health for adult malaria vector control has been reported across Côte d’Ivoire [8], limiting the country’s malaria vector control toolkit. Côte d’Ivoire was the first West African country to report the knockdown resistance (*kdr*) mutation in the 1990s, before it was detected all over the continent [9, 10]. Moreover, due to the intense exposure of *Anopheles* mosquitoes to insecticides used in both agriculture and public health, *An. gambiae* s.l. have developed resistance in several parts of the country [8, 11, 12].

A first attempt to manage insecticide resistance using ITNs was the development of ITNs that incorporate a pyrethroid and a synergist, piperonyl butoxide (PBO), which has been shown to increase the mortality of malaria vectors with metabolic resistance involving mono-oxygenases. Currently, five ITNs types with PBO have been developed, all of which received WHO pre-qualification and are available on the market. Three of these are treated with a combination of PBO and deltamethrin (PermaNet 3.0, Tsara Plus, Tsara Boost), one with PBO and permethrin (Olyset Plus), and one with PBO and alpha-cypermethrin (Veeralin) [13–15]. The effectiveness of these ITNs in a given area depends on the extent of the involvement of metabolic resistance mediated by mono-oxygenase enzymes in vector populations. A countrywide distribution of such nets therefore requires baseline entomological studies to show the involvement of enzyme activities in pyrethroid resistance of *An. gambiae* s.l. and the effect of the synergist in restoring susceptibility of the targeted vector population to pyrethroids [16].

Recent research exploring other classes of insecticides identified chlorfenapyr-based tools as a promising option for vector control. Furthermore, WHO Pre-Qualification (WHO PQ) has approved chlorfenapyr-based nets and additional studies are ongoing under the UNITAID-funded New Nets Project to demonstrate the public health impact of those nets for wide use. The different mode of action of chlorfenapyr is expected to be effective in the presence of pyrethroid resistance developed by mosquitoes through several mutations [17, 18]. The oxidative removal of the *N*-ethoxymethyl group of the pro-insecticide chlorfenapyr leads to a toxic form of the molecule identified as CL 303268 which functions to uncouple oxidative phosphorylation and disrupt metabolic pathways of ATP production in mitochondria [19]. This molecule has low mammalian toxicity and is classified as a slightly hazardous insecticide by the WHO. Due to its novel mode of action, chlorfenapyr is unlikely to show any cross-resistance with standard neurotoxic insecticides as observed in *Anopheles* mosquitoes [20, 21]. Chlorfenapyr has been evaluated in laboratory bioassays and in experimental hut studies for ITNs in several countries including Côte d’Ivoire, Burkina Faso, Tanzania, and for both ITNs and IRS in Benin [22–26]. Results from those studies suggest that chlorfenapyr is promising as a new molecule for ITNs and IRS to control populations of pyrethroid-resistant malaria vectors [27, 28].

With regards to widespread resistance to the insecticides previously used and the aim of introducing new generation vector control tools in Côte d’Ivoire, ITNs with chlorfenapyr and PBO represent options to overcome and manage widespread resistance. The current study was undertaken within a countrywide insecticide resistance monitoring effort to support the National Malaria Control Programme (NMCP) in making vector control decisions in Côte d’Ivoire and also to contribute to the knowledge base for chlorfenapyr more broadly.

## Methods

### Study sites

**Fig. 1:**
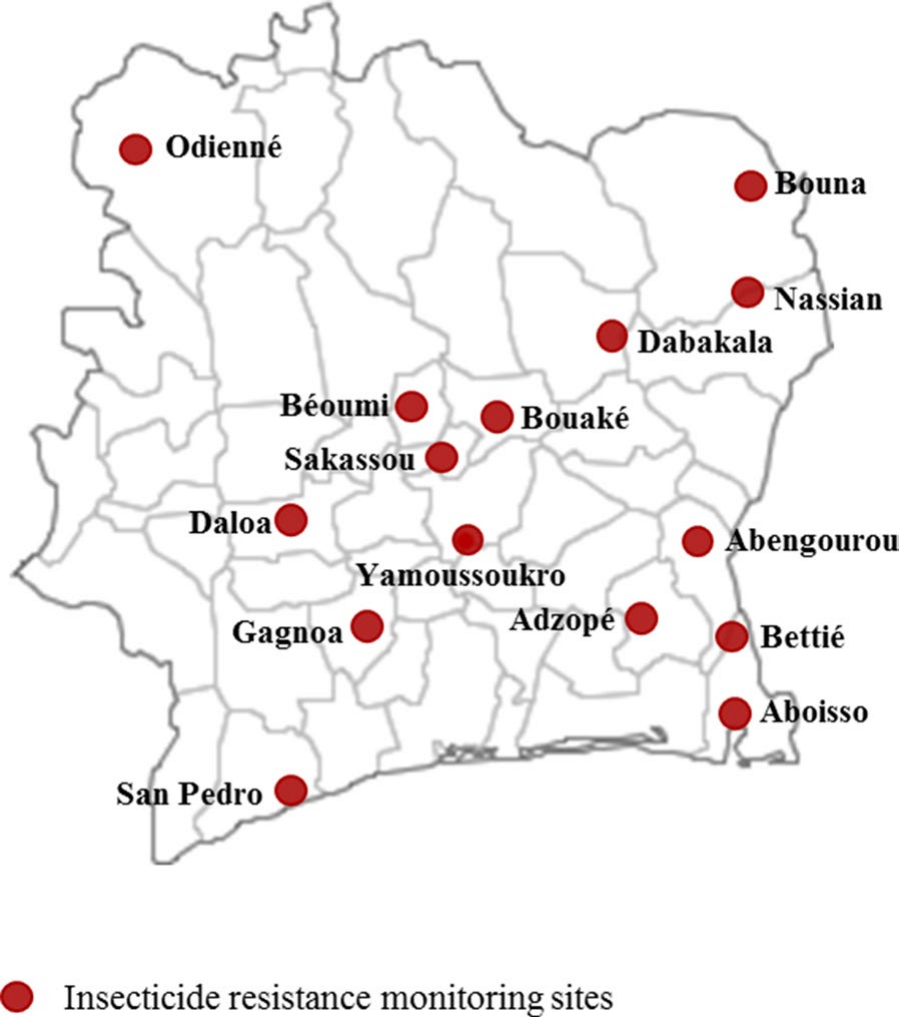
Map of the study sites

Côte d’Ivoire is divided into four ecological zones including the forest zone in the South and West, the transitional zone in the Centre, the savanna zone and the Sudan savanna zone in the North. The climate in the southern forest zone is equatorial, with annual rainfall between 2100 mm and 2500 mm and subequatorial in the western forest with a range of average rainfall between 1600 and 2300 mm per year. In the central transitional zone of the country, the climate is tropical, with an average of 1200 mm rainfall per year. The climate is also tropical in the northern savanna and Sudan Savanah zone, with an annual average rainfall of 900 mm. Insecticide resistance surveys were carried out in fifteen sites distributed across the country, including Aboisso, Adzopé, and San Pedro in the South, Bouaké, Béoumi, Dabakala, Sakassou and Yamoussoukro in the Centre, Odienné in the North-West, Bouna and Nassian in the North-East, Daloa and Gagnoa in the west and Abengourou and Bettié in the East (Fig. 1). Of Those sites, ten represented the NMCP sentinel sites were all malaria control activities and impact were monitored all year round. The additional sites (Bettie, Gagnoa, Sakassou, Dabakala and Béoumi) were surveyed based on their malaria endemicity and targeted for indoor residual spraying site selection. Furthermore, the country has a history of two mass campaigns of pyrethroid-only long lasting insecticide treated net (LLIN) distributions conducted in 2014 and 2017.

The study was conducted between June and September 2019. *Anopheles gambiae* s.l. larvae and pupae were collected from several urban and rural larval habitats in each site using the dipping method, pooled and reared to adulthood in a field laboratory at each site.

### Insecticides and synergist

Papers impregnated with deltamethrin (0.05%, 0.25% and 0.5%), permethrin (0.75%, 3.75% and 7.5%), alpha-cypermethrin (0.05%, 0.25% and 0.5%) and PBO (4%) were obtained from University Sains Malaysia (USM).

Pre-weighed preparations of technical-grade active ingredient of chlorfenapyr (BASF Corporation USA, Batch N 2130H070HV) provided by the Centers for Disease Control and Prevention (CDC), Atlanta, were used to prepare 50 mL of two doses (100 µg (a.i)/bottle and 200 µg (a.i)/bottle) by adding the prescribed volume of acetone solvent. The solution obtained was vortexed to ensure that the solution was uniformly mixed. All insecticide solutions and papers were kept at 4 °C before and after each test.

Prior to the field susceptibility testing, two impregnated papers received were randomly selected from each box and tested against a laboratory-maintained susceptible strain of *An. gambiae* Kisumu to verify the effectiveness of the paper. Both chlorfenapyr solutions (100 µg (a.i.)/bottle and 200 µg (a.i.)/bottle) were also tested using the CDC bottle assay against *An. gambiae* Kisumu to check the efficacy on a susceptible population of mosquitoes.

### WHO susceptibility test, intensity and synergist assays

Insecticide susceptibility tests were conducted using WHO tube tests on 2–4 day old adult female *An. gambiae* s.l. [29, 30]. The diagnostic concentrations of deltamethrin (0.05%), permethrin (0.75%), alpha-cypermethrin (0.05%) were tested alone and in combination with PBO concurrently in all sites. For the synergist assays, mosquitoes were pre-exposed to PBO for one hour before being exposed to the pyrethroid insecticides for one hour. Resistance intensity at five and ten times the diagnostic concentration of deltamethrin, permethrin and alpha-cypermethrin was also tested in each site using WHO susceptibility kits. Two tubes lined with silicone oil treated papers were set in parallel during each test and served as controls. The start and end temperatures during all tests were recorded at 9 sites, and Hobo data loggers (HOBO UX100-003, Onset Computer Co., Bourne, MA, USA) were used to track temperatures every 10 min in the laboratory in two sites (Beoumi and Sakassou) (Additional file 1).

### CDC bottle assay with chlorfenapyr

Due to non-availability of a WHO recommended protocol for testing chlorfenapyr, two doses (100 µg (a.i.)/bottle and 200 µg (a.i.)/bottle) were selected based on a literature review [21, 27, 28, 31] and used to coat the CDC bottles. The bottles were coated following the protocol described by Brogdon et al. [32], with 1 mL of chlorfenapyr diluted in acetone at the concentration of 100 µg (a.i.)/bottle and 200 µg (a.i.)/bottle. The coated bottles were wrapped in aluminum foil and dried overnight at room temperature. A modified protocol was designed following the procedures described by Brogdon et al. [32]. Fifteen to 20 adult mosquitoes (2–4 days old) that emerged from field collected larvae were aspirated into 250 mL chlorfenapyr-coated Wheaton bottles for 1 h, after which the mosquitoes were released into a clean cage, aspirated back to paper cups and fed with 10% sugar solution. Mortality was recorded at 24, 48, and 72 h after exposure. Two bottles coated with acetone only were prepared similarly to serve as controls.

### Species identification of the *An. gambiae* s.l. population

A subsample of about fifty *An. gambiae* s.l. mosquitoes was randomly selected among the population tested per site and processed for molecular identification of the species of the *An. gambiae* complex following the SINE PCR protocol described by Santolamazza et al*.* [33]. Additionally, the presence of knocked-down resistance (*kdr*)-West and East resistance mechanisms was characterized within each *An. gambiae* s.l. population using the Taqman protocol described by Bass et al. [34] and the presence of acetylcholinesterase (*ace*-1) gene was determined following the protocol described by Weill et al. [35]*.*

### Statistical analysis

Insecticide resistance status was defined following WHO criteria [27] with corrected mortality after 24 h for pyrethroids and 72 h for chlorfenapyr < 90% as confirmed resistance, between 90 and < 98% as possible resistance, and ≥ 98% as susceptible. Mortality was corrected using Abbott’s formula when the mortality of the control tubes was above 5% and less than 20%.

Corrected mortality of:

- 98–100% at 5× the diagnostic dose indicates low resistance intensity
- Less than 98% at 5× diagnostic dose implies testing the 10× diagnostic dose
- 98–100% at 10× the diagnostic dose confirms a moderate resistance intensity.
- Less than 98% at 10× the diagnostic doses indicates high resistance intensity.

For the synergist assays, an increase in the mortality after pre-exposure to PBO compared to the diagnostic dose of the insecticide alone indicates the involvement of enzymes such as P450s in the population tested. The mortality of mosquitoes exposed to PBO + pyrethroid was also compared with that of the insecticides alone and plotted using Graph Pad Prism 5 software. The total percentage mortality of all sites against each insecticide alone and those of the PBO + insecticides were analyzed and compared using a Chi-squared test with Stata 14 (Stata Corporation, College Station, Texas, USA).

## Results

### WHO susceptibility test, intensity and synergist assays

**Fig. 2:**
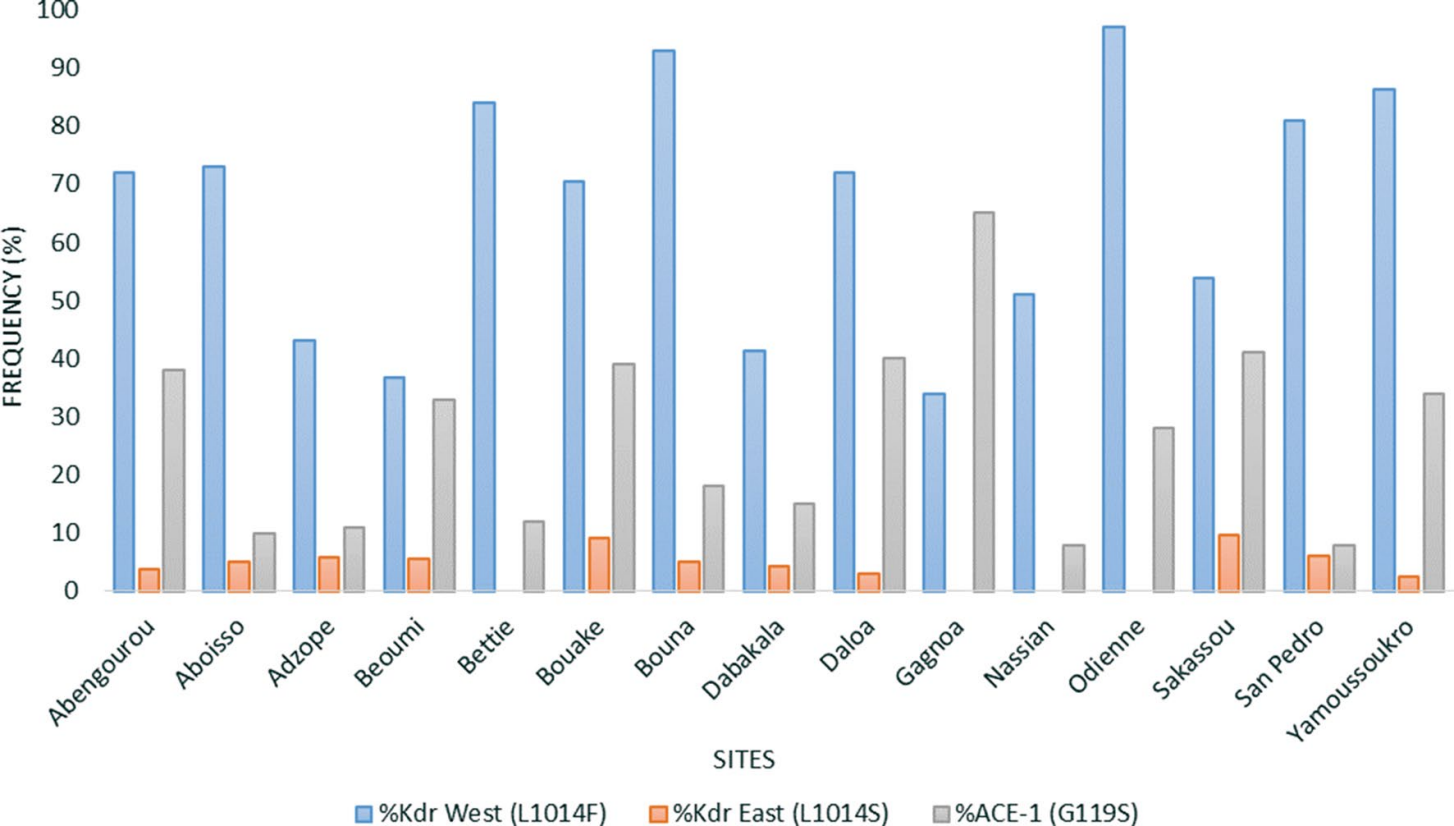
Distribution and frequency of the knocked-down resistance (*kdr*) West, *kdr*-East and acetylcholinesterase (*ace*-1) within the *An. gambiae* s.l. population of each site

The *An. gambiae* s.l. population tested was primarily composed of *Anopheles coluzzii* (> 95%) in Abengourou, Adzopé, Béoumi, Bettié, Dabakala, Daloa, Gagnoa, Sakassou and San Pedro, while *An. gambiae *sensu stricto (s.s.) was the only member of the complex found in the country northern sites: Bouna, Nassian and Odienné. However, both species were in sympatry (≈ 50% each) in Aboisso, Bouaké and Yamoussoukro. Furthermore, high *kdr*-West resistance allele frequency was recorded in all the 15 sites ranging between 34% in Gagnoa and 97% in Odienné. The *kdr*-East was also observed in few sites but at a lower frequency compared to the *kdr*-West. The highest frequency was observed in Sakassou with 10% *kdr*-East resistance allele frequency. The *ace*-1 frequency was relatively high in eight of the fifteen sites (between 28% in Odienné to 65% in Gagnoa) (Fig. 2).

Full susceptibility (100% mortality) was recorded for all insecticides tested against *An. gambiae* Kisumu and no sign of compromised quality and/or efficacy of the insecticide impregnated papers and the chlorfenapyr-coated bottles were observed.

The start temperatures in the 9 sites where start and end temperatures were recorded had a mean of 25.4 °C (minimum 23.0 °C, maximum 28.2 °C) at the start of tests and 25.2 °C at the end of tests (minimum 23.8 °C, maximum 26.8 °C). During the tests with chlorfenapyr, the range was smaller, with mean start temperatures of 25.6 °C (minimum 24.0 °C, maximum 26.7 °C) and end temperatures of 25.2 °C (minimum 24.0 °C, maximum 26.0 °C). As would be expected since the loggers measured the temperature every 10 min, the Hobo loggers picked up a larger variation in temperature, with laboratories ranging from 22.9 to 28.3 °C (Beoumi) and 24.1 °C to 28.4 °C (Sakassou).

**Fig. 3:**
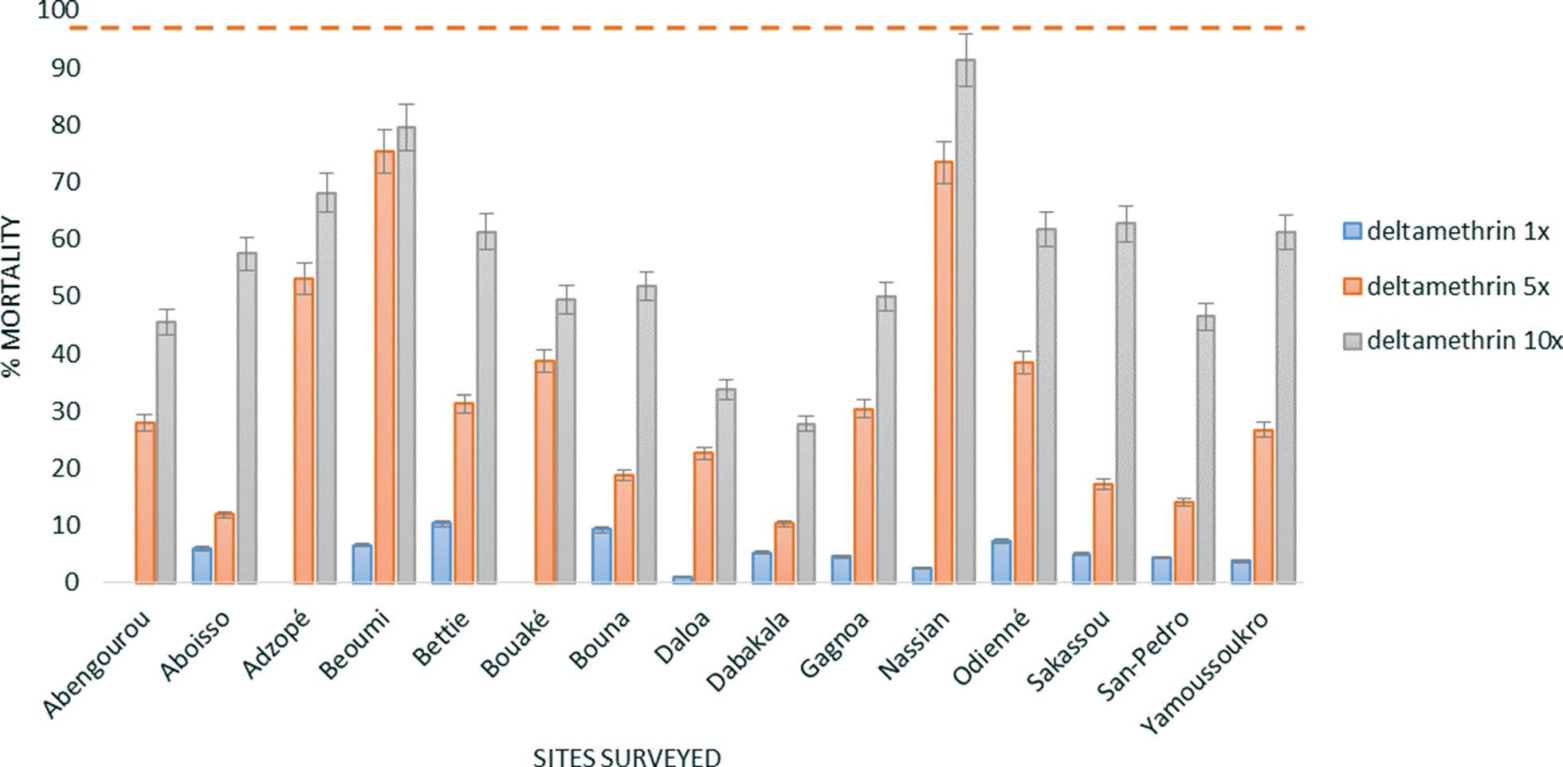
WHO susceptibility and intensity assays of deltamethrin against *An. gambiae* s.l. of the different sites surveyed. Error bars represent the standard errors at the Y axis and the red line represents the susceptibility treshold

**Fig. 4:**
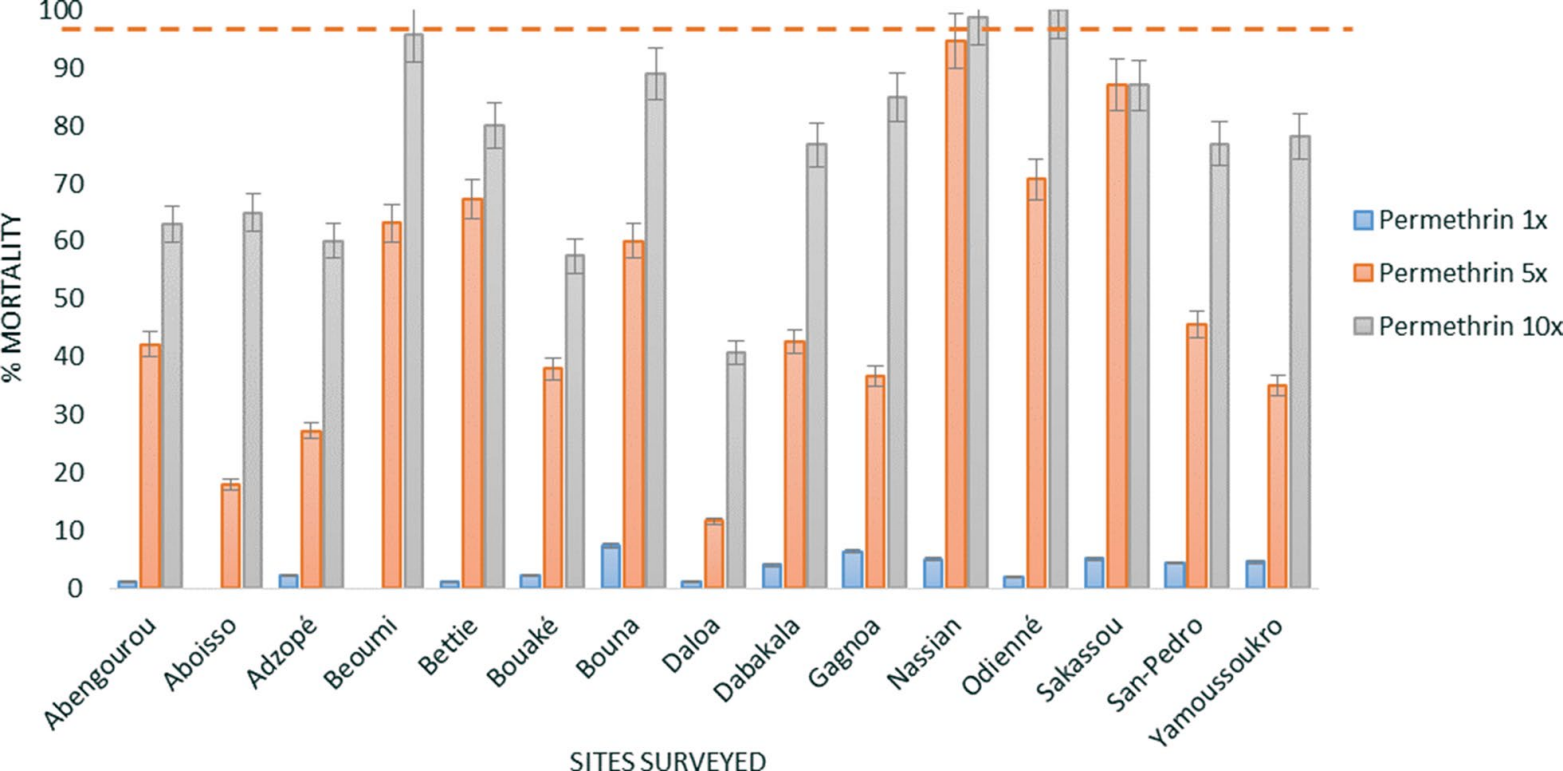
WHO susceptibility and intensity assays of permethrin against *An. gambiae* s.l. of the different sites surveyed. Error bars represent the standard errors at the Y axis and the red line represents the susceptibility treshold

**Fig. 5:**
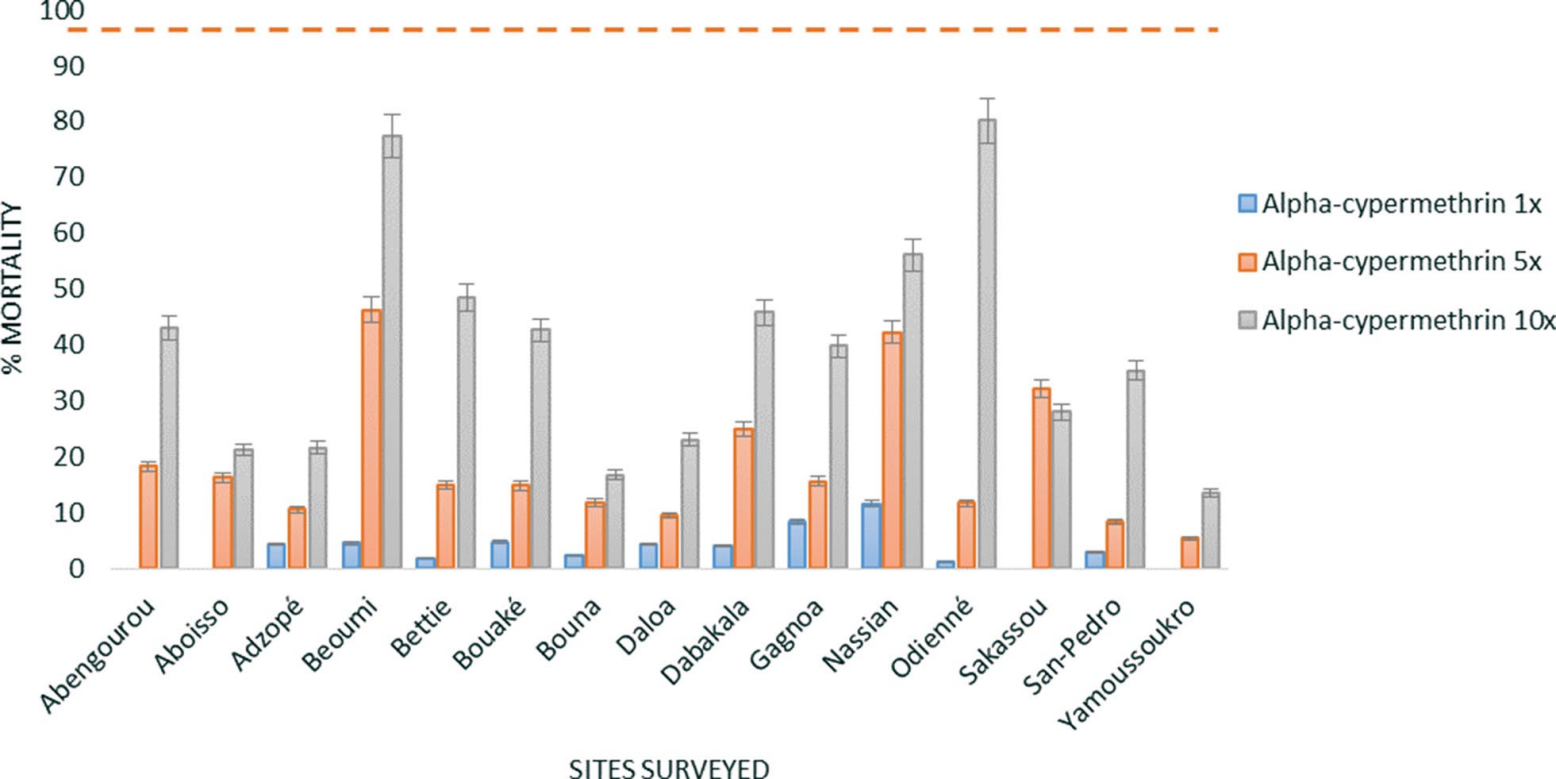
WHO susceptibility and intensity assays of alpha-cypermethrin against *An. gambiae* s.l. of the different sites surveyed. Error bars represent the standard errors at the Y axis and the red line represents the susceptibility treshold

Resistance of *An. gambiae* s.l. mosquitoes to pyrethroids at diagnostic doses was observed in all fifteen sites surveyed. The mean mortality rate (all sites) was below 10% for all three pyrethroids: 4.4% (± 0.8 standard error (SE) for deltamethrin, 3.1% (± 0.6 SE) for permethrin and 3.4% (± 0.9 SE) for alpha-cypermethrin (Figs. 3, 4, 5). Additionally, high pyrethroid resistance intensity was observed in all sites with survival recorded at 10× the diagnostic doses (DD) of deltamethrin (between 27.8% mortality at Dabakala and 79.5% at Beoumi), permethrin (between 40.7% mortality at Daloa and 95.9% at Beoumi) and alpha-cypermethrin (between 13.5% mortality at Yamoussoukro and 80.2% at Odienné). Only *An. gambiae* s.l. collected from Odienné (100% mortality at 10× DD) and Nassian (98.9% mortality at 10× DD) showed moderate resistance intensity to permethrin.

**Fig. 6:**
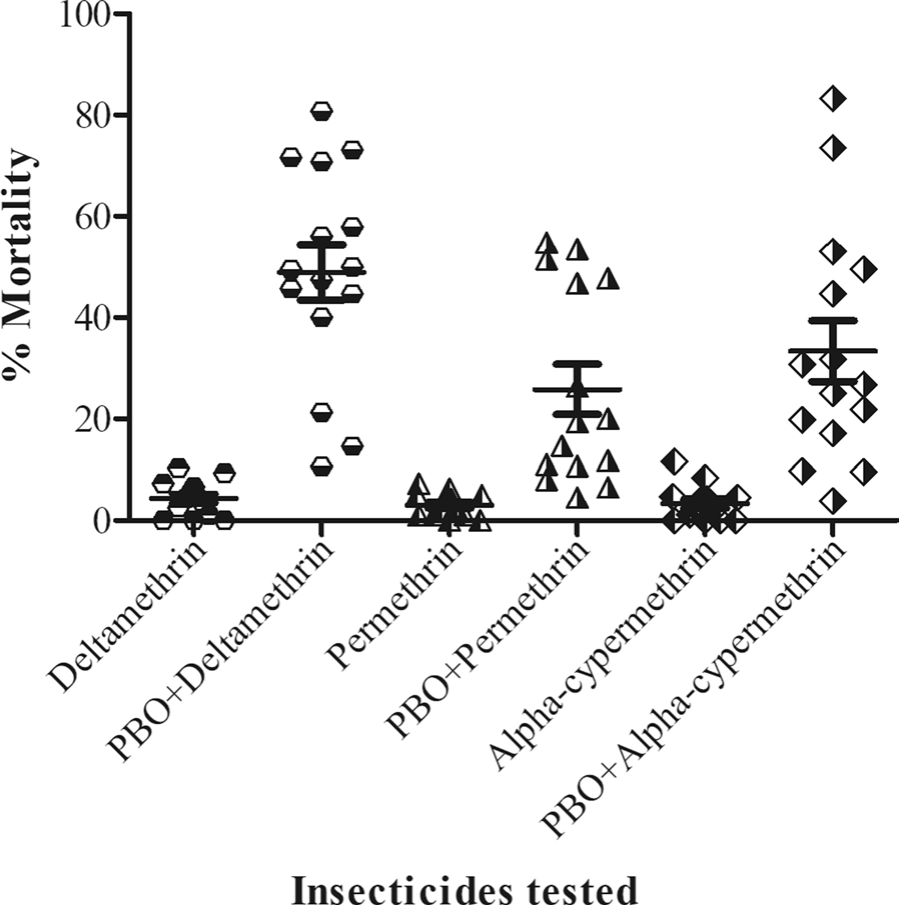
Comparative percentage increment in susceptibility of *An. gambiae* s.l. using the synergist piperonyl butoxide (PBO) + pyrethroids in relation to pyrethroids alone across all sites surveyed. Significant increment in susceptibility was observed with the addition of PBO (p < 0.001) for all three pyrethroids

When all sites were considered together, synergist assays using PBO pre-exposure yielded statistically significant increases in the mean mortality for all three pyrethroids (p < 0.0001) (Fig. 6; Additional file 2). Average permethrin mortality increased from 2.9% when tested alone to 26% mortality with PBO pre-exposure. Deltamethrin mortality was 4.3% mortality without PBO and 47.7% mortality with PBO pre-exposure. Alpha-cypermethrin mortality was 3.3% without PBO and 33.7% with PBO pre-exposure (Additional file 3).

### Chlorfenapyr CDC bottle assay

Susceptibility (98–100%) was recorded for chlorfenapyr after 24 h and 48 h delayed mortality for the dose of 200 µg/bottle and 100 µg/bottle, respectively, when tested against the *An. gambiae* Kisumu strain.

**Fig. 7:**
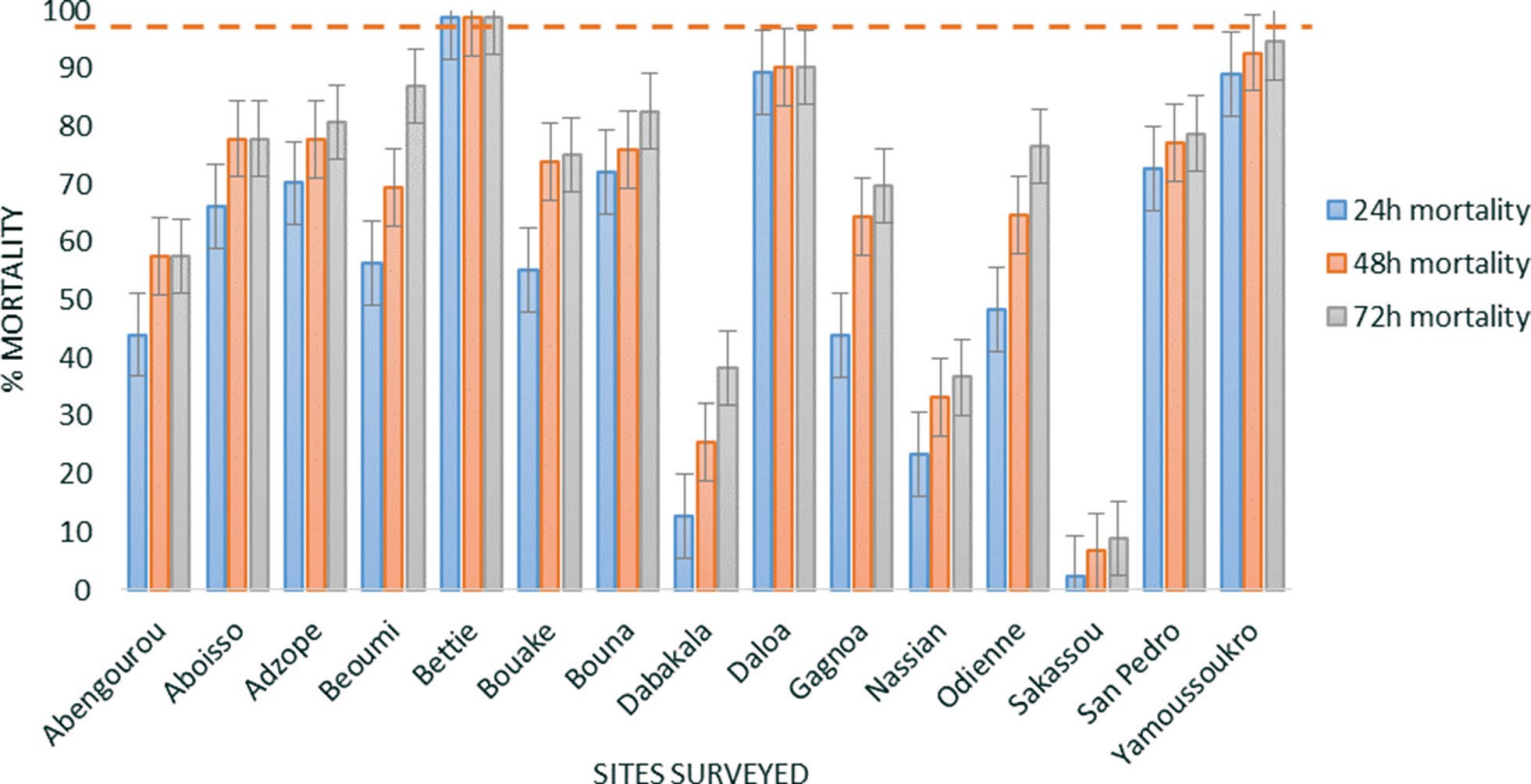
CDC bottle assay using chlorfenapyr 100 µg/bottle against *An. gambiae* s.l. of the different sites surveyed. Error bars represent the standard errors at the Y axis and the red line represents the susceptibility treshold

**Fig. 8:**
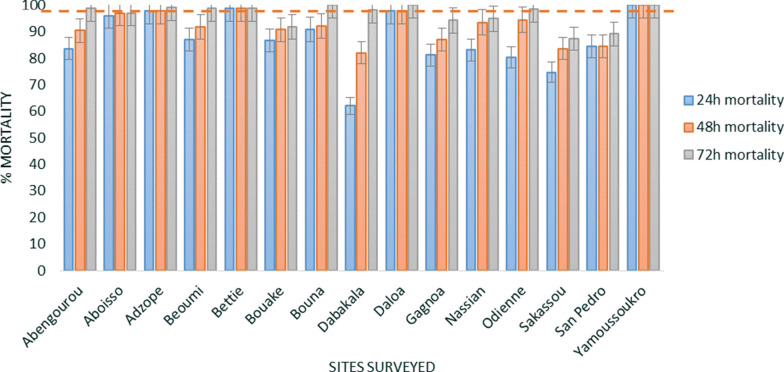
CDC bottle assay using chlorfenapyr 200 µg/bottle against *An. gambiae* s.l. of the different sites surveyed. Error bars represent the standard errors at the Y axis and the red line represents the susceptibility treshold

*Anopheles gambiae* s.l. from only one out of 15 sites (Bettie) recorded susceptibility (98.8% mortality) when exposed to the 100 µg/bottle dose of chlorfenapyr (Fig. 7). The 200 µg/bottle dose yielded more than 98% mortality in ten out of 15 sites. Mortality ranging between 87.3% in Sakassou to 94.8% in Nassian was observed at the dose of 200 µg/bottle in Bouake, Gagnoa, Nassian, Sakassou, and San Pedro (Fig. 8) (Additional file 4).

## Discussion

Resistance of *An. gambiae* s.l. mosquitoes to pyrethroids observed in this study is consistent with the previously described trends in Côte d’Ivoire involving various and high insecticide resistance mechanisms such as the *kdr*-West and East and the *ace-*1 mutations [8, 11, 12, 36, 37]. Testing the intensity of the resistance from 15 sites showed that resistance intensity to all three pyrethroid insecticides is high. This presents a challenge for malaria control as it limits the country’s options for insecticide-based vector control interventions. The level of resistance observed could be attributed to the different applications of insecticide in the country, either for agriculture or public health. Agriculture accounts for 24% of GDP in Côte d’Ivoire, one of the largest producers and exporters of coffee, cocoa, cashew, and palm oil worldwide. A recent survey by the ministry of agriculture revealed that more than 20,000 tons of chemicals including pesticides and insecticides were applied in the country in 2018 for agricultural purposes including 40% of this amount entering illegally [38]. This likely contributed to the increasing resistance of malaria vectors to insecticides commonly used for insect control in general and malaria vector control specifically [36, 37, 39]. In Côte d’Ivoire, the two major malaria vectors include *An. gambiae* and *An. coluzzii* [40]. Both species generally breed in fresh water pools resulting from human activities such as irrigated rice fields and vegetable gardens, which are often treated with insecticides [36, 37, 39, 41]. As described by Chouaibou et al. [42], agricultural pesticides contain several insecticide molecules and particularly pyrethroids and carbamates, which contribute to the increased and widespread resistance of the vectors [39]. Furthermore, studies conducted in Western and Eastern Africa have shown a significant increase in the frequency of genes associated with pyrethroid resistance immediately following the implementation of an ITN campaign [7]. Moreover, the National Strategic Plan (NSP) developed in 2018 by the Côte d’Ivoire National Malaria Control Programme (NMCP) prioritized universal ITN coverage in households across the country through mass distribution campaign of pyrethroid-only ITNs.

In this study, pre-exposure to PBO before the different pyrethroids tested showed statistically significant but still insufficient increases in the mortality of the *An. gambiae* s.l. populations. These results would support the use of PBO-treated vector control tools in select areas where significant reversal of resistance was observed after pre-exposure to PBO. In some locations, PBO has resulted in increased mortality of even highly resistant mosquito populations [43]. Out of several combinations of PBO ITNs, combinations with the type II pyrethroids (deltamethrin and alpha-cypermethrin) showed higher performance than those with the type I pyrethroid (permethrin), consistent with several other studies comparing the different types of PBO ITNs [44, 45]. These data could inform the selection and stratification of the PBO ITNs for resistance management in Côte d’Ivoire.

Higher mortality of mosquitoes was observed against chlorfenapyr particularly in areas of probable metabolic resistance where higher mortality was noted after pre-exposure of the mosquitoes to PBO before the different pyrethroids. It is known that significant increase in mortality after exposure to PBO indicates the involvement of P450 enzymes in the insecticide resistance of the vectors. The trends observed were consistent with previous observations, when assessing the lethality of chlorfenapyr in areas of high metabolic resistance in *Aedes aegypti* after mosquitoes are exposed or not to PBO [17].

Chlorfenapyr is a protoxin requiring activation, via cytochrome P450 monooxygenases, to exert its toxic effects, via uncoupling of oxidative phosphorylation [17, 19]. The expression in *An. gambiae* s.l. of some cytochrome P450s involved in oxidative metabolism are under circadian control and more strongly expressed at night when *Anopheles* flight and host-seeking activity are higher [46, 47]. Such a model could suggest that exposure at night could likely produce higher mortality results compared to what was observed using traditional testing methods. The supposition that laboratory-based exposures to technical grade active ingredient or any other formulation where behaviours are excluded, likely negates attributes of oxidative uncoupling by chlorfenapyr when compared to host-seeking behaviours which include free-flying physiologically heightened states known to enhance pro-insecticide chlorfenapyr intoxication to mosquitoes as demonstrated by Oxborough et al. [31].

Furthermore, previous work showed that testing completed during the night induces significantly greater mortality than daytime bioassays, indicating the possibility of higher effectiveness of chlorfenapyr-based ITNs such as Interceptor® G2 since the peak biting hours of the vectors are typically late in the night [31]. Moreover, several studies are still ongoing to understand and delineate the appropriate diagnostic doses and times for testing chlorfenapyr susceptibility [31, 48, 49]. This study will therefore contribute to the evidence base for supporting the determination of an appropriate diagnostic dose. With regards to the data generated, in ten out of 15 sites where the tests were conducted, 98% or greater mortality was recorded at the dose of 200 µg/bottle against *An. gambiae* s.l., indicating that chlorfenapyr could be considered as an option to manage the resistant population of malaria vectors in Côte d’Ivoire, where resistance has continuously increased over the past several decades.

Several trials and studies have been conducted on the Interceptor® G2 net. As described by Bayili et al., Camara et al. [22, 23] and Ngufor et al. [26] chlorfenapyr-treated nets evaluated in several areas with documented pyrethroid resistance have been proven effective for controlling pyrethroid-resistant malaria vectors and could contribute to malaria control decision-making and insecticide resistance management in Côte d’Ivoire.

Though it yielded important findings, this study had limitations. There is no standardized and WHO approved test protocol available for chlorfenapyr. The best available information was used to determine the concentration of insecticide tested, exposure and holding period, and test conditions and interpretation of the result. The protocol might be optimized by conducting susceptibility tests at different temperatures, comparing tests results conducted during day light versus night, or putting the mosquitoes in dark room during exposure period. Until such standardized protocol is developed for guidance, the data showing reduced susceptibility of *An. gambiae* s.l. to chlorfenapyr shall be interpreted with caution applying it only to specific study conditions. Despite this limitation, the information gathered could contribute to informing vector control programming in Côte d’Ivoire.

## Conclusion

As resistance to all pyrethroid insecticides is very high across the country, new tools such as chlorfenapyr- and PBO-combination ITNs may be appropriate for Côte d’Ivoire. This study demonstrated the relative increase in effectiveness observed when exposing the pyrethroid-resistant mosquitoes to either PBO in combination with pyrethroids or to chlorfenapyr. Both options represent avenues for many African country NMCPs to develop insecticide resistance management strategies. In Côte d’Ivoire, Interceptor® G2, a chlorfenapyr- and alpha-cypermethrin-based ITN could therefore be considered for a stratified distribution campaign in addition to PBO ITNs. The data gathered across the country within this study could also support the determination of the diagnostic concentration for testing the susceptibility status of *An. gambiae* s.l. against chlorfenapyr while the molecule is still being tested for appropriate concentrations in ITNs and IRS.

## Supplementary Information

**Additional file 1** WHO susceptibility tests and CDC bottle assays testing conditions (Temperature and humidity) of the different sites surveyed.

**Additional file 2** Insecticide susceptibility test and resistance intensity data and graphs.

**Additional file 3** Synergist assays using pyrethroid insecticides and piperonyl butoxide data.

**Additional file 4** Chlorfenapyr assay test data and graphs.

## Data Availability

All data generated or analyzed during this study are included in this published article and its Additional files.

## Abbreviations

Ace-1: Acetylcholinesterase
BASF: Badische Anilin- & Soda-Fabrik
CDC: Centers for Disease Control and Prevention
IRS: Indoor residual spraying
ITN: Insecticide-treated net
Kdr: Knocked-down resistance
NMCP: National Malaria Control Programme
NSP: National Strategic Plan
PBO: Piperonyl butoxide
PCR: Polymerase Chain Reaction
PQ: Prequalification
SE: Standard Error
SINE: Short Interspersed Element
USM: University Sains of Malaysia
WHO: World Health Organization
