# Supplementary figures and images for: Susceptibility of Anopheles gambiae from Côte d’Ivoire to insecticides used on insecticide-treated nets: evaluating the additional entomological impact of piperonyl butoxide and chlorfenapyr
Source: Malar J. 2020 Dec 9;19:454. doi: 10.1186/s12936-020-03523-y (PMC7725118; doi:10.1186/s12936-020-03523-y)

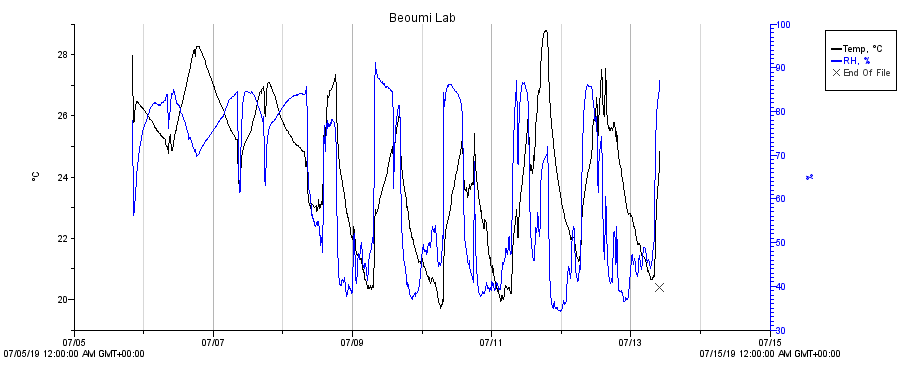


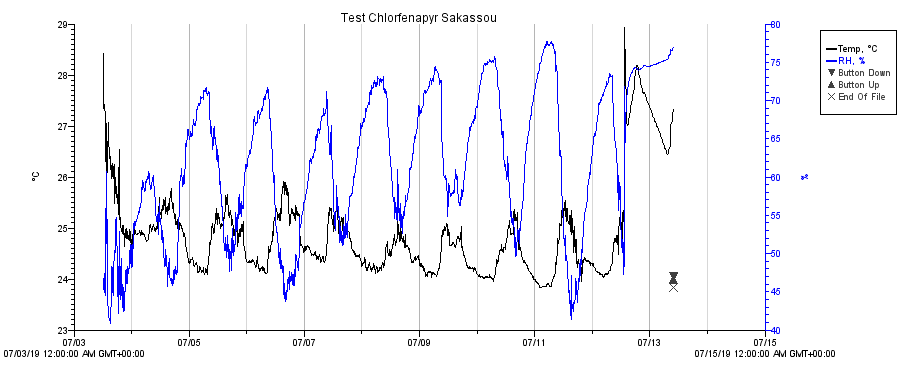

Supplement: Supplementary file 4 — Additional file 4 Chlorfenapyr assay test data and graphs. [file 12936_2020_3523_MOESM4_ESM.zip › Testing Temperature/graph Beoumi-Sakassou.docx]
